# RETRACTION: Genetic Association between Asthma and Alopecia Areata: A Two‐Sample Mendelian Randomization Study

**DOI:** 10.1111/srt.70248

**Published:** 2025-10-08

**RETRACTION**: W. Du, K. He, X. Liu, T. Yin, S. Xiao, and Y. Zheng, “Genetic association between asthma and alopecia areata: A two‐sample Mendelian randomization study,” *Skin Research Technology* 30, no. 7 (2024): e13844, https://doi.org/10.1111/srt.13844.

The above article, published online on 4 July 2024 in Wiley Online Library (wileyonlinelibrary.com), has been retracted by agreement between *Skin Research and Technology*; and John Wiley & Sons Ltd.

The article was accepted for publication following an insufficient peer review process that does not meet the standards of the journal's peer review policy. As a result, the findings reported in the article cannot be regarded as scientifically sound, particularly as the article does not adequately present the biological rationale necessary to support the valid application of Mendelian Randomization to the research question. Accordingly, the editorial team cannot vouch for the validity of the methodological approach employed, nor the reliability of the study's conclusions. Therefore, the journal and the publisher have made the decision to retract the article. The authors have been informed of this decision and disagree with it.

